# Fiber Network Formation in Semi-Flexible Polymer Solutions: An Exploratory Computational Study

**DOI:** 10.3390/gels4020027

**Published:** 2018-03-22

**Authors:** Fernando Vargas-Lara, Jack F. Douglas

**Affiliations:** Materials Science and Engineering Division, National Institute of Standards and Technology, Gaithersburg, MD 20899, USA

**Keywords:** semi-flexible polymer, fiber, network, gels, persistence length, rigidity

## Abstract

The formation of gels through the bundling of semi-flexible polymer chains into fiber networks is ubiquitous in diverse manufactured and natural materials, and, accordingly, we perform exploratory molecular dynamics simulations of a coarse-grained model of semi-flexible polymers in a solution with attractive lateral interchain interactions to understand essential features of this type of gel formation. After showing that our model gives rise to fibrous gels resembling real gels of this kind, we investigate how the extent of fiber bundling influences the “melting” temperature, $T_{m}$, and the emergent rigidification of model bundled fibers having a fixed number of chains, *N*, within them. Based on our preliminary observations, we suggest the fiber size is kinetically selected by a reduced thermodynamic driving force and a slowing of the dynamics within the fibers associated with their progressive rigidification with the inclusion of an increasing number of chains in the bundle.

## 1. Introduction

Gels are the epitome of soft matter and these materials arise from a diverse range of polymeric and nanoparticle substituent materials [1]. However, the engineering of gels to fully exploit their properties for numerous industrial [2] and medical applications [3,4] requires a better understanding of the process of gel formation [5], factors that influence the gel stability, and the relations between gel structures and the unique rheological properties of this class of materials.

Gelation encompasses diverse processes in which polymers and particles mutually interact to become localized in space, leading to the emergence of macroscopic rigidity [6]. This amorphous solidification process can occur both as an equilibrium or a non-equilibrium process, and both as a thermally reversible or irreversible process. In accordance, there are diverse types of gels [7]. The present work focuses on a type of gelation that arises rather ubiquitously in suspensions of semi-flexible polymers with mutual attractive interpolymer interactions [6]. Such systems characteristically exhibit a tendency to bundle and to form networks due to bundle branching [6], although branching is not necessary for gelation if the fibers are very long [8]. The diameter of these bundles, $D_{b}$, is often rather uniform in many molecular fiber-forming systems where the fiber diameter typically ranges from a few nm to a few tens of nm [9,10,11,12,13]. In the present work, we introduce a minimal computational model to explore network formation in semi-flexible polymer suspensions with attractive intermolecular interactions. First, we show by molecular dynamics simulations that our model gives rise to a network formation with a morphology similar to that found in experimental observations on this type of gel, including the tendency to form fibers with a near-uniform diameter, and which is naturally influenced by the stiffness of the isolated semi-flexible polymers and the strength of the mutual interaction between the polymer chains.

**Figure 1:**
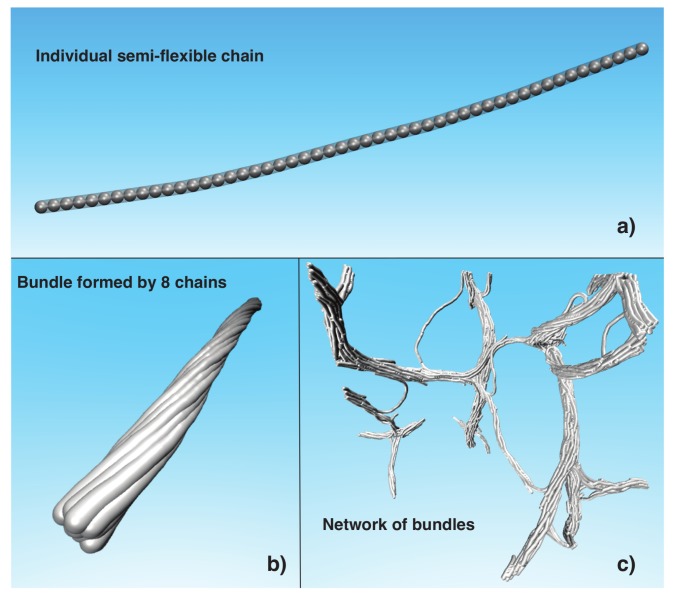
(**a**) Representative configuration of semi-flexible chain formed by L=50σ connected beads; (**b**) Twisted bundle of eight chains with L=100σ; (**c**) Representative configuration of network of bundles formed by 50 initially dispersed chains with length L=100σ.

To gain insight into the thermal stability of this type of gel, which appears to “melt” upon heating, we consider the melting of ideal fiber bundles composed of *N* polymer chains (see Figure 1). The melting temperature, $T_{m}$, of these bundles initially increases sharply with *N*, reflecting the increased interchain interaction strength between the chains, but $T_{m}$ saturates to a finite value for large *N*. It occurred to us that this phenomenon might be related the increase of the bending stiffness (κ), of the bundle with increasing *N*, and we confirmed this hypothesis in our computational model where we found that κ likewise first increases strongly with *N*, but then saturates to a finite value of large *N*. Moreover, we found that the magnitude of *N* at which this saturation occurs is comparable to the average number of chains, N=10±3, in a typical network fiber, formed after a thermal quench of the semi-flexible polymer solution. Based on these observations, we suggest that the average fiber diameter, $D_{b}$, is controlled by two effects: a progressive decrease in the enthalpy of binding per chain within the fiber bundles with increasing *N*, and a concomitant increase in the fiber stiffness with increasing *N*. Together, these thermodynamic and dynamic fiber characteristics inhibit the unlimited the growth of diameter fibers. We can get an order of magnitude estimate of these fibers by considering typical molecular dimensions of assembling molecules. The diameter of the cellulose molecule is about 1.5 nm or somewhat larger if a hydration layer is included, and fiber former proteins, such as actin, typically have a diameter of about 5 nm to 6 nm. If we then take the molecular diameter of our individual chain in our simulation to be σ≈2 nm to 6 nm, then the expected bundle diameter, $D_{b}$, is on the order of $D_{b}∼O(10$ nm).

## 2. Results and Discussion

### 2.1. Calculation of Bundle Characteristic Rigidity

To explore how the characteristic rigidity of the bundles depends on chain structural parameters and the number of chains within the chain bundles, we perform a systematic study of the bundle persistence length, $l_{p}$, of model bundles containing a *fixed* number of chains, *N* (see Figure 1b). In particular, we vary the chain length, *L*, the chain intrinsic rigidity, $k_{\mathrm{bend}}$, and chain cohesive interaction strength, ε.

#### 2.1.1. Effect of Individual Chain Stiffness on Fiber Bundle Stiffness

We start by calculating the persistence length of an individual isolated chain, $l_{\mathrm{pc}}$, by calculating the average projection of the chain end-to-end distance, $R_{e}$, on the first bond of the chain, $l_{1}$ [14,15],

(1) $$l_{\mathrm{pc}}=\langle R_{e}\cdot l_{1}\rangle /\langle l_{1}\rangle .$$

We calculate the persistence length of a bundle formed by *N* chains, $l_{p}\left(N\right)$, by computing by the average value of the persistence length,

(2) $$\langle l_{p}\rangle =\left(l_{p1}+l_{p2}+l_{p3}+\ldots +l_{\mathrm{pN}}\right)/N.$$

Here, $l_{\mathrm{pi}}$ represents the persistence length of the *i*-chain forming the bundle. Additionally, we define the relative bundle persistence length, $l_{\mathrm{pr}}\left(N\right)$, by the ratio

(3) $$l_{\mathrm{pr}}\left(N\right)=\langle l_{p}\rangle /l_{\mathrm{pc}}$$

#### 2.1.2. Effect of the Chain Stiffness on the Bundle Rigidity

**Figure 2:**
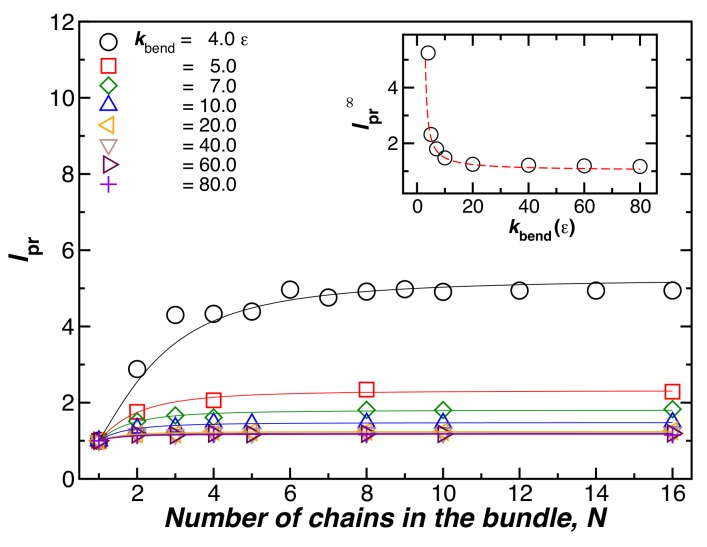
The relative bundle persistence length, $l_{\mathrm{pr}}$, as a function of the number of chains in the bundle, *N*. The chain bending parameter, $k_{\mathrm{bend}}$, is varied. The chain length, L=100σ, and T=0.1ε are fixed. The inset shows the plateau values of $l_{\mathrm{pr}}^{\infty }$ for large *N*, obtained by fitting the data to Equation (4). We observe that $l_{\mathrm{pr}}^{\infty }$ decreases when $k_{\mathrm{bend}}$ increases, so that the increase in stiffness upon binding is smaller than the increase in stiffness in the individual chains.

Figure 2 shows the bundle persistence length $l_{\mathrm{pr}}$ relative to the individual chains for bundles with a fixed length L=100σ, and T=0.1ε, as a function of the chain bending energy, $k_{\mathrm{bend}}$. This ratio quantifies the extent of rigidification that occurs from fiber formation. The symbols in Figure 2 are numerical estimates and the lines are fits to the empirical crossover relation,

(4) $$l_{\mathrm{pr}}=\frac{l_{\mathrm{pr}}^{\infty }\;N^{2}}{l_{\mathrm{pr}}^{\infty }-1+N^{2}},$$

where $l_{\mathrm{pr}}$ is the plateau value of $l_{\mathrm{pr}}$ when *N* is large. The initial $N^{2}$ scaling for small *N* means that $l_{\mathrm{pr}}$ initially increases linearly with the square of the cross-sectional diameter of the fiber [16], the scaling expected from the continuum theory of uniform fibers (see our discussion below). In the inset of Figure 2, we consider the influence of the bending energy of the chains on the stiffness of the resulting fibers when *N* is fixed. We see that the relative value of the persistence length, $l_{\mathrm{pr}}^{\infty }$, decreases when $k_{\mathrm{bend}}$ is increased, and that the degree of stiffness increases monotonically with *N*, the effect saturating at large *N*.

#### 2.1.3. Effect of the Chain Length *L* on Fiber Bundle Rigidity

**Figure 3:**
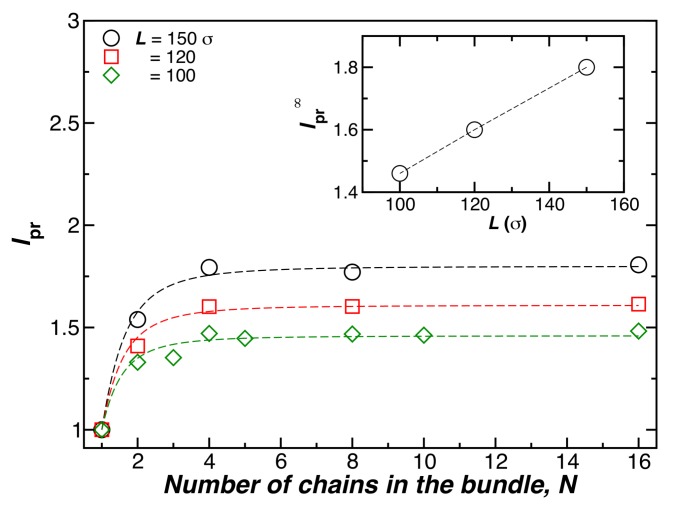
The relative bundle persistence length, $l_{\mathrm{pr}}$, as a function of the number of chains in the bundle *N*, where L=100σ and T=0.1ε. Here, the chain length (L=100σ) and temperature (T=0.1ε) are fixed.

Figure 3 shows $l_{\mathrm{pr}}$ for bundles formed by *N* chains having fixed length (L=100σ), at a fixed temperature (T=0.1ε), and a fixed chain bending stiffness ($k_{\mathrm{bend}}=10.0\;\varepsilon$). The symbols show simulation estimates of $l_{p}$, and the lines are fits of this data to Equation (4). Increasing the chain length also increases $l_{p}$ and the chain rigidity, as observed in our simulations [15], and experiments [17] on duplex DNA and tubulin [18]. We expect this effect to ultimately saturate for large *L*, but this expected “long fiber” regime has not been reached in our simulations. The dotted lines in the main part of Figure 3 are again shown as fits to Equation (4).

#### 2.1.4. Effect of the Chain Attractive Interaction on the Bundle Rigidity

**Figure 4:**
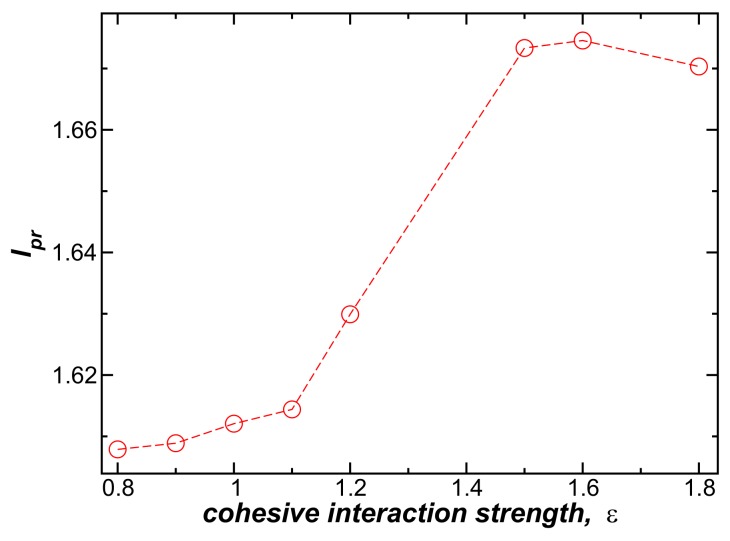
The normalized bundle persistence length, $l_{\mathrm{pr}}$, as a function of attractive interaction strength, ε, for bundles in which N=4, L=100σ, and $k_{\mathrm{ben}}=7.0\;\varepsilon$ at T=0.1ε.

To study the influence of the interchain interaction strength ε on the bundle persistence length, $l_{p}$, we vary ε in the range, (0.8≤ε≤1.8). Increasing ε initially increases $l_{p}$, and thus the bundle rigidity, but the effect saturates when ε becomes large. We likewise found that increasing the interaction strength between model DNA chains increases the persistence length of duplex DNA [15], where the interaction-induced rigidification saturates for large ε, as in our fiber bundle data shown in Figure 4. In the context of duplex DNA, this effect was interpreted in terms of a *coupling* of the elastic distortion upon binding and associated enhanced cohesive interaction between the chains, and we suggest the same phenomenon is operative in our fiber networks. The intermolecular interaction strength acting between the polymer chains within the fibers is evidently an important parameter in this type of gel, as further indicated in recent experimental studies of fiber-forming gels [19]. The strength of the cohesive interaction has also been shown to correlate strongly with the occurrence of fiber network gel formation [10,20].

### 2.2. Melting of Model Fiber Bundles

**Figure 5:**
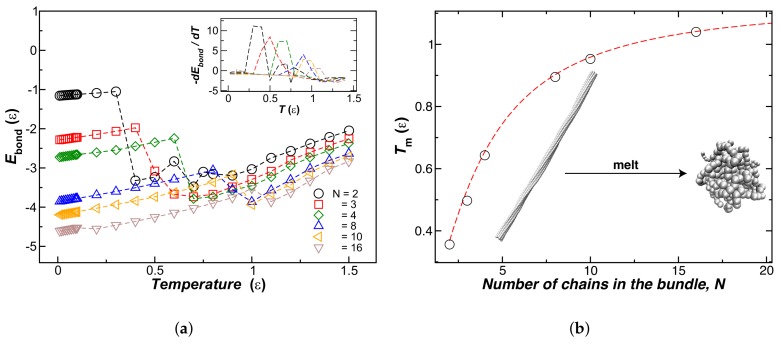
(**a**) Normalized bonded energy $E_{\mathrm{bond}}\left(T\right)=U_{\mathrm{bond}}\left(T\right)/N_{p}$ for bundles formed by different number of chains *N*. The inset shows the first derivative of $-E_{\mathrm{bond}}$ with respect to *T* as a function of *T*. We identify the melting bundle–globule transition temperature, $T_{m}$, as the peak position of the fitted function; (**b**) shows $T_{m}$ as a function of *N* for chains with fixed length (L=100σ) and fixed $k_{\mathrm{bend}}=7.0\;\varepsilon$. The insets are representative of an eight-chain bundle at a temperature above and below $T_{m}$.

We next explore bundle thermodynamic stability by increasing the temperature of thermal equilibrated chain bundles in the temperature range 0.01≤T≤1.5. To achieve this characterization, we compute the normalized bonding energy, $E_{\mathrm{bond}}=U_{\mathrm{bond}}\left(T\right)/N_{p}$, as a function of *T* for bundles with a fixed number of chains *N* within the bundle, and results for $E_{\mathrm{bond}}$ are shown in Figure 5a. Unexpectedly, we found the bundles first exhibit a “melting” transition into globular structures before separating into individual strands at higher temperatures. The location of this melting transition can be determined by calculating the first derivative of $E_{\mathrm{bond}}\left(T\right)$ with respect to *T*, illustrated in the inset of Figure 5a. We define $T_{m}$ as the temperature at which the fiber bundle transforms into a globule. Figure 5b shows our $T_{m}$ estimates as a function of *N*. We observe that $T_{m}$ at first increases with *N*, and then saturates to a constant value, an evidently repeating behavior.

**Figure 6:**
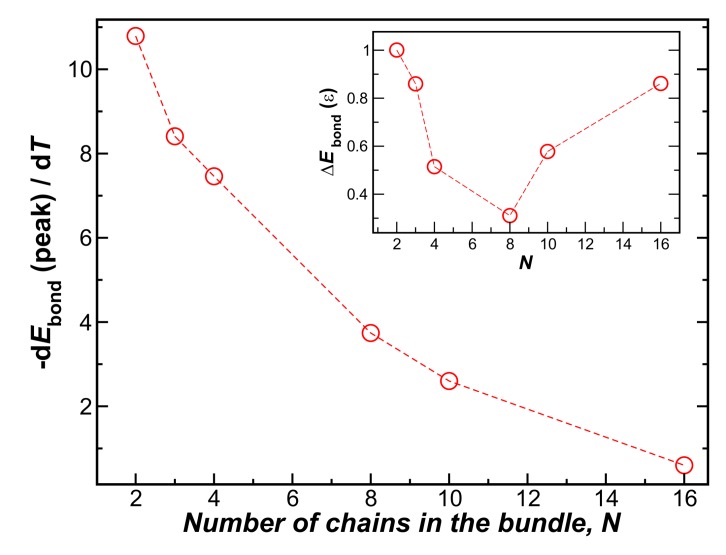
The peak value of the first derivative of the bundle energy at the melting transition temperature, $T_{m}$, as a function of the number chains in the bundle, *N*. The inset shows the relative change in energy, $\Delta E_{\mathrm{bond}}=\left|E_{\mathrm{bond}}\left(T=0\right)-E_{\mathrm{bond}}\left(T=T_{m}\right)\right|$, which exhibits a minimum near N≈8. The energetic driving force per chain evidently rapidly decreases with *N*; the enthalpy change upon melting is minimized for N≈8, a value on the order of the observed average number of chains in the non-equilibrium fiber network bundles (see Figure 1c).

Unsurprisingly, larger bundles melt at a higher temperature, as normally found for nanocrystals, but there is a limit to this effect. On the other hand, the inset shows that the differential change of energy per chain upon binding, $dE_{\mathrm{bond}}\left(T\right)/dT$, at $T_{m}$ decreases sharply with *N*. In Figure 6, we plot the peak values of this energy derivative as a function of the number of chains in the bundle, where we see that this quantity approaches 0 for N≈15. The inset to Figure 6 also shows the overall change of the chain enthalpy at the transition, relative to the bundle energy at low temperature. This measure of the enthalpy change upon melting evidently becomes minimized for *N* at about N≈8. The change of enthalpy upon bundling per chain rapidly decreases for N∼O(10), a scale consistent with the fiber bundle average size found in the non-equilibrium fiber networks (see Section 4.2).

We next offer a tentative interpretation of the origin of the fixed average fiber diameter. Apparently, the rate of growth of the fibers slows down abruptly after some point since the enthalpy change per chain upon adding polymers the fiber becomes progressively small with increasing *N*, resulting in metastable fibers having a well-defined average diameter. The twisting on the chains in the bundles appear to play a significant role in this general trend. The more flexible the fiber, the more readily the chains within it can twist to achieve more interchain contacts, thus lowering the enthalpy per chain. At some value of *N*, the fiber becomes so rigid that the chains within the fiber lose their capacity to twist so that this mechanism of lowering the energy per chain is attenuated [21]. Claessens et al. [22] and and Bruss and Grayson [23] have likewise recently suggested that an interplay between fiber twisting and lateral intermolecular interactions regulates bundle diameter in fiber networks. There have also been interesting arguments recently suggesting that the diameter of self-assembled nanofibers arises from packing frustration [24,25]. We do not see how these mechanisms of fiber formation apply to our fiber networks. At the same time, we appreciate that multiple distinct mechanisms might give rise to fibers having a nearly fixed diameter.

### 2.3. Temperature Dependence of Bundle Persistence Length

**Figure 7:**
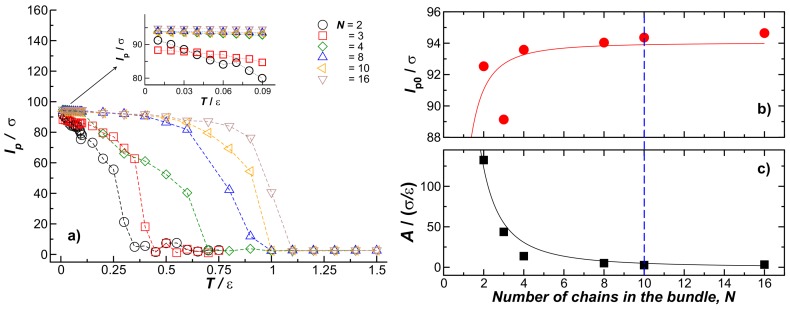
(**a**) The bundle persistence length, $l_{p}$, as a function of *T* for bundles formed by *N* fibers indicated in the figure legend. The inset shows $l_{p}$ in the low temperature regime; (**b**,**c**) $l_{p0}$ and *A*, respectively. Both parameters were obtained by fitting $l_{p}$ in the low temperature regime to a linear relationship.

Figure 7 shows the *T* dependence of the bundle persistence length $l_{p}$ for N=(2,3,4,8,10,16,32) chains within the bundles. Here the chains have fixed length and chain stiffness, L=100σ and $k_{\mathrm{bend}}=7.0\;\varepsilon$, respectively. The *T* dependence of the bundle persistence length ($l_{p}\left(N\right)$) is evidently dominated by the fiber melting process so that its *T* variation is quite unlike the predictions of the worm-like chain model. In particular, we see that increasing *N* leads to an increase of $l_{p}\left(N,T\right)$ that progressively weakens with increasing *N*, while an increase of fiber rigidity increases the persistence length without limit in the worm-like chain mode, particularly since $l_{p}$ for a continuum fiber scales as $l_{p}∼\kappa /T$, where κ is the fiber-bending stiffness. It is well known that κ scales as the bending moment, *I*, times the Young’s modulus, *Y*, of the fiber, κ, so that κ should scale as $\kappa_{0}∼N^{2}$, where $\kappa_{0}$ is the fiber stiffness of an individual chain within the fiber [26]. This scaling results from the scaling of a circular fiber with a radius *r*, as $∼\;r^{4}$ [26] since the fiber and the radius scale as $r\;∼N^{1/2}$ when *N* becomes large.

The *T* dependence of $l_{p}$ shown in Figure 7a can be described by the phenomenological expression for the Young’s modulus of the fiber, *Y*, that is widely applicable to ordinary crystalline materials [27],

(5) $$l_{p}\left(N,T\right)=l_{p0}-A\;T\;\exp\left(-T_{D}/T\right),$$

where $l_{p0}$ is the persistence length value in the limit of low temperature, $l_{p0}\left(T\to 0\right)$, and *A* and $T_{D}$ are *N*-dependent fitting parameters, given a theoretical interpretation in terms of the Gruneisen parameter (a measure of crystal anhamonicity) and the Debye temperature, respectively, by Anderson [28]. To obtain $l_{p0}$ and *A* with reasonable confidence, we consider the low temperature regime shown as an inset in Figure 7b. In this low-*T* regime, it is clear that the dominant terms in Equation (5), $l_{p}\left(N,T\right)=l_{p0}\left(N\right)-A\;T$ provide a good approximation. Figure 7b shows the values of $l_{p0}\left(N\right)$ estimated from fits in the low-*T* regime; we see that the fiber persistence length in the low temperature limit follows the same trend as in Figure 2, where *T* was fixed to T=0.1ε. As noted above, when *N* is small, we find a scaling consistent with the continuum theory of worm-like fibers, $l_{p0}∼N^{2}$, but this stiffening effect with increasing *N* ultimately saturates, i.e., N∼O(10). Figure 7c shows *A* as a function of *N*, where *A* saturates for N∼O(10). In this figure, we note that $A∼N^{-2}$, that is a trend completely contrary to expectations of the worm-like chain model where *A* is predicted to increase with fiber rigidity. A diminished anharmonic interaction strength provides a natural interpretation of the decrease of *A* with *N*. The *T* dependence of $l_{p}\left(N,T\right)$ decreases with increasing *N*, an effect associated with an upward shift in the melting temperature with increasing *N*. Approximate $N^{2}$ scaling has been observed in actin bundles [29] while $l_{p}$ for collagen fibers has shown a regime in which $l_{p}$ is insensitive to fiber diameter [30], so there seems to be some fragmentary experimental evidence supporting the trend indicated in Figure 7. Further experimental and computational studies are warranted, however, to better understand this counter-intuitive phenomenon.

## 3. Conclusions

We have investigated the formation of fiber networks from molecular dynamics simulations of initially dispersed semi-flexible chains with attractive interchain interactions that favors the formation of fibrous bundles of polymeric chains. After verifying that our model gives rise to the formation of fibrous networks composed of bundled polymers with a relatively uniform diameter, along with branching defects, we then sought to understand the emergent rigidification and thermodynamic stability of model fibers of *N* chains and the factors that lead to a saturation of the fiber diameter in this type of network formation.

Our exploratory simulations indicate that the relative persistence length $l_{\mathrm{pr}}$ saturates to a constant value of N≈8 with increasing numbers of chains within the bundle having a fixed interchain interaction strength, ε, and length, *L*. This saturation value of *N* is reasonably consistent with the *N* value found in the fibers formed in the formation of a non-equilibrium fibrous networks, N∼O(10) and this phenomenon is consistent with the order of magnitude of the diameter of fibers observed experimentally. We also find that increasing the strength of the cohesive interaction parameter, ε, also increases the fiber bundle persistence length somewhat, but this increase likewise saturates for a sufficiently large ε. Chain rigidity and intermolecular cohesive interaction strength are evidently key physical parameters governing chain bundling.

The melting of the fiber bundles is more complicated than we initially expected. We find that bundle persistence length drops sharply as the fibers start to unbind, but the lateral attractive interactions between the semi-flexible polymers at first lead to the formation of a droplet-like structures rather than dispersed polymers, although at very high *T* the chains ultimately dissociate into solution. The melting temperature shows a tendency to saturate for a large number of chains *N* within the bundles N∼O(100) so that there is no obvious link between the saturation in $l_{p}$ with increasing *N*, discussed above, and the fiber melting temperature, $T_{m}$. On the other hand, we can see that changes in enthalpy change per chain slow down rapidly with increasing *N*, leading to a decrease of the thermodynamic driving force for more chains to be incorporated into the bundles. We tentatively suggest that this thermodynamic effect leads to a kinetic bottleneck that selects out bundles with a definite average diameter in fiber networks formed under non-equilibrium temperature-quench conditions. We also observe that the chain persistence length $l_{p}$ saturates for a similar *N* value, i.e., N≈8. Based on our previous experience of melting DNA [15], where a saturation in $l_{p}$ with inter-base interaction strength ε and the chain length was observed, we propose that the tendency of the fibers to exhibit a limited diameter derives both from a sharp drop in the enthalpy per chain and progressive rigidification upon forming bundles. There are both thermodynamic and kinetic factors leading to a kinetic bottleneck that results in fiber-diameter formation being self-limiting in the degree of chain association.

## 4. Materials and Methods

### 4.1. Computational Model

We utilize molecular dynamics (MD) simulations of a semi-flexible chain coarse-grained model to explore the chain bundling and networks formed by semi-flexible chains, as well as the bundle thermodynamic stability upon varying temperature. In this model, we represent each semi-flexible chain as a set of connected “beads” (gray spheres in Figure 1a [31]). The soft core–excluded volume interaction among all the beads is given by the Weeks–Chandler–Andersen potential ($U_{\mathrm{WCA}}$),

(6) $$U_{\mathrm{WCA}}=U_{\mathrm{LJ}}\left(r\right)-U_{\mathrm{LJ}}\left(r_{c}\right)-\left(r-r_{c}\right)\frac{dU_{\mathrm{LJ}}\left(r-r_{c}\right)}{dr}{|}_{r_{c}},$$

where $U_{\mathrm{LJ}}$ is the 12-6 Lennard-Jones potential, and ε and σ are the LJ energy and length parameters, respectively. The distance between two beads is given by *r*, and $r_{c}$ is a cutoff distance. All the beads interact attractively since $r_{c}=2.5\;\sigma$. The “beads” of the polymer are connected by a finitely extensible, non-linear elastic (FENE) anharmonic spring potential,

(7) $$U_{\mathrm{FENE}}\left(r\right)=-\frac{k_{F}R_{0}^{2}}{2}\ln\left[1-{\left(\frac{r-r_{s}}{R_{0}}\right)}^{2}\right].$$

where $k_{F}=30\;\varepsilon /\sigma^{2}$ and $R_{0}=1.5\;\sigma$. We control chain stiffness by using a three-body angular potential,

(8) $$U_{\mathrm{bend}}\left(\theta \right)=k_{\mathrm{bend}}\left(1+\cos\theta \right),$$

where the potential strength $k_{\mathrm{bend}}$ can take the values $k_{\mathrm{bend}}=\left(4,5,7,10,20,40,60,80\right)\;\varepsilon$. We consider chains with chain lengths L=(100,120,150,160)σ. To study thermodynamic stability of the bundles, we generate bundles formed by N=(1,2,3,4,5,8,10,16,32) semi-flexible chains. Figure 1b shows a representative image of a bundle formed by eight chains. For the rest of the paper, $N_{p}$ refers to the total number of beads in the bundle, so that $N_{p}=NL$.

All our simulations are performed in a canonical ensemble ($N_{p}VT$) with temperature *T* in the range $\left(0.1\leq T\leq 1.5\right)\;\varepsilon /k_{B}$, where $k_{B}$ is the Boltzmann constant, and for simplicity, we choose reduced units, where $k_{B}=1.0$, *T* and energy are given in ε units, and length is given in units of σ. We control *T* by using the Nosé–Hoover method [32,33]. To study the bundle stability, we run MD simulations for periods of $\geq 10^{8}$ time steps to ensure all systems have reached thermal equilibrium, or at least achieving a steady state condition whose properties are not aging at a detectable rate. We then compute average properties for three randomly initiated sets of $2\times 10^{6}$ thermal equilibrated configurations. All our MD simulations were performed using the Large-scale Atomic/Molecular Massively Parallel Simulator (LAMMPS) [34] using periodic boundary conditions and we render the images of Figure 1 using the Visual Molecular Dynamics (VMD) program [35].

### 4.2. Exploratory Study of the Formation of Fiber Networks

To study the average diameter of the bundles, $D_{b}$, formed in the network, we consider model chain solutions with 50 chains, where each chain is formed by L=100σ, and $k_{\mathrm{bend}}=0.7\;\varepsilon$, corresponding to systems with the volume fraction $\phi =2.61\times 10^{-3}$. We quench the 12 copies of randomly generated systems using three values of cooling rate of ($10^{-7},10^{-5},10^{-4}$) (1/time steps) for temperatures in the range of (0.1≤T≤2)ε. Figure 1c shows a rendered image of a network formed using the protocol described above. We found that the average bundle diameter, $D_{b}$, derived from our model of fiber assembly lies in a broad range, $(7\leq D_{b}\leq 13)\;\sigma$. Smaller fibrils apparently arise from “tie-chains” that span distinct fiber bundles, creating branch points in the network. The conformations of these tie chain bundles have a rather distorted shape and their diameters are normally smaller than in the main bundles. In the next sections, we attempt to understand the origin of the nearly “fixed” average diameter, $D_{b}$, of the bundles in the network and the thermodynamic stability of the fiber network. Animations of the process fiber network formation are given in the Supplementary Materials.

## Supplementary Materials

The Supplementary Materials are available online at http://www.mdpi.com/2310-2861/4/2/27/s1.
